# Traumatic Tympanic Membrane perforation: An aetiological profile

**DOI:** 10.1186/1756-0500-2-232

**Published:** 2009-11-21

**Authors:** Olushola A Afolabi, Shuaib K Aremu, Biodun S Alabi, S Segun-Busari

**Affiliations:** 1 University of Ilorin/University of Ilorin Teaching Hospital, PO Box 931, Ilorin, Nigeria

## Abstract

**Background:**

Traumatic perforation of the tympanic membrane may be due to direct or indirect source. The aim of the study is to profile the various aetiologies of traumatic tympanic membrane perforation in Ilorin, north central Nigeria.

A retrospective review of 64 patients seen at the University of Ilorin Teaching hospital, Ilorin, Nigeria over a ten year period (January 1998 to Dec 2007) with history of traumatic tympanic membrane perforation from various causes, these also included multiply injured patients with bleeding from middle ear as part of their presentations. The data retrieved included the biodata, the clinical presentations, source of injury, the clinical findings and the treatment outcome. The data were entered into an SPSS version 11 computer soft ware and analyzed descriptively.

**Findings:**

Sixty four (64) ears were analysed, Age range 6 months to 50 yrs, mean age of 29.2 yrs 7.9% of them were ≤5 years, 29.7% between 21-34 years, and 37.7% were 35 years and above. The male to female ratio was 2.5:1.0. Commonest aetiology was from slaps, then road traffic injury (RTI) in 35.9% and 23.5%, Majority of the slap injury were from fights (30.5%), security agents, senior students and cultists at schools (17.4% each). Sudden hearing loss was a typical presentation (95.3%), majority of the patient defaulted from follow up once the symptoms of bleeding and pain subsided. Only 7.8% had neomembrane formation on follow up

**Conclusion:**

Traumatic perforation of the tympanic membrane is an uncommon injury that is under-reported, there is the need to educate on alternative punitive measure among students and security agents, unskilled removal of foreign body, early identification, evaluation and referral of patients reduces the attendant morbidity.

## Background

Trauma generally is blight on our society and it is a major cause of morbidity and mortality in any society [1]. This could be in form of assaults, road traffic injury, domestic, industrial and sports injuries. These are relatively on the increase in our society although it is difficult to know the economic impact on Nigeria, however it is estimated that the annual cost of dealing with this tragedy is more than $100 billion in the USA.

In a 1999 study, it was found that the average personal injury in the workplace costs more than $8,000 in lost earnings [2]. Trauma patients consume more health care resources than heart and cancer patients combined, and whereas mortality from heart disease and cancer is declining, the incidence from trauma is increasing [3,4].

Trauma to the ear could be simple blunt trauma to the pinna; laceration of the pinna avulsion of part or the whole of the pinna; uncomplicated tympanic membrane perforation; dislocation of the ossicles; longitudinal and transverse fractures of the petrous temporal bone with associated loss of inner ear and facial nerve function [5-11]

Trauma to the tympanic membrane and the middle ear can be caused by overpressure (slap, fight, assault from security agents and road traffic injury (RTI)), thermal or caustic burns, blunt or penetrating injuries such as instrumentations and barotraumas [12,13]. Overpressure is by far the most common mechanism of trauma to the tympanic membrane [12]. Traumatic perforation of the tympanic membrane may be caused by direct impact of fluids and direct pressure from outside. The aim of the study is to profile the various aetiologies of traumatic tympanic membrane perforation

## Methods

This is a retrospective review of 64 patients seen at the Ear, Nose and Throat clinic and the accident and emergency unit of the University of Ilorin Teaching hospital, Ilorin, Nigeria over a ten year period between January 1998 to Dec 2007 with history of bleeding from the ear due to trauma from various causes, also included were patients with multiple trauma who also had traumatic tympanic membrane perforation as part of the presentation. The data retrieved included the biodata, the clinical presentation, source of injury, the clinical findings and the outcome of the patients were entered into an SPSS version 11.0 computer soft ware and analyzed descriptively.

## Results

Seventy patients (70) were found to have traumatic tympanic membrane perforation however 6 were excluded because of incomplete data thus only 64 were analyzed and formed the basis for this study. Age range 6 months to 50 yrs with a mean age of 29.2 yrs and modal age of 35 years. About 5 (7.9%) of them were ≤5 years and majority of the patients were between 35 and 50 years of age Table 1. There are 46 (71.9%) males and 18(28.1%) females with a male to female ratio of 2.5:1 and cross tabulation and predisposition male were mostly affected in most of the aetiologies except in fall where no male was recorded table 2

**Table 1:** Age distribution of patients with traumatic TM perforations

Age in years	Frequency (%)
6 months-5 yrs	5 (7.9%)
6 years-10 yrs	1 (1.6%)
11-20 yrs	15 (23.5%)
21-34 yrs	19 (29.7%)

35-64 yrs	24 (37.7%)

**Table 2:** Predisposition and sex of patients

Predisposition	Frequency	
	**Male**	**Female**
Slap	15	8
Instrumentation	4	3
Self during ear cleaning	5	2
RTI	9	1
Foreign body	10	3
Explosion	3	
Fall		1

Total	46	18

The commonest aetiology recorded was from slaps, then road traffic injury (RTI) in 35.9% and 23.5% respectively table 3

**Table 3:** Aetiological profile of TM perforations.

Aetiology	Frequency (%)
Slaps	23 (35.9)
Instrumentations	7 (10.9)
Self Ear cleaning	7 (10.9)
Road Traffic Injury	15 (23.5)
Foreign body	8 (12.5)
Explosions	3 (4.7)
Falls	1 (1.6)

Total	64 (100.0)

Majority of the slap injury were from fights, security agents, senior students and cultists at schools in 30.5%, 17.4% and 17.4% respectively (Table 4).

**Table 4:** Sources of Slaps

Sources of slap	Frequency (%)
Security Agent	4 (17.4)
Assault from Fight	7 (30.5)
Spouse	2 (8.7)
Armed Robbery	3 (13.0)
Senior student/cultists	4 (17.4)
Sibling	3 (13.0)

Total	23 (100.0)

Traumatic tympanic membrane perforation showed that 36 left ears and 28 right ears were affected.

Majority of the patients (95%) had associated sudden hearing loss, tinnitus in 52% while 24 (37.5%) of the patient had progression to chronic suppurative otitis media (Table 5) and it was observed that majority of the patient defaulted from the follow up once the symptoms of bleeding and pain subsided mostly after an average of three follow up visits. Out of the few that came for follow up only 7.8% had neomembrane formation.

**Table 5:** Clinical presentation of the patients

Presenting Symptoms	Frequency (%)
Hearing loss	61 (95.3)
Bleeding	44 (68.8)
CSF leak	10 (15.6)
Dizziness	6 (9.4)
Tinnitus	33 (52)
Infection	24 (37.5)

Pain	38 (59.4)

## Discussion

The tympanic membrane (TM) is an important component of sound conduction as its vibratory characteristic is necessary for sound transmission in human beings [14].

Trauma to TM and the middle ear can be caused by overpressure, thermal or caustic burns, blunt or penetrating injuries, and barotraumas [12,13]. Overpressure is by far the most common mechanism of trauma to the TM in our study similar to various studies else where [5,12,13].

Traumatic tympanic membrane affects all age groups with a mean age of 29.2 yrs similar to a study from the south-eastern part of Nigeria that had a mean age of 27.6 yrs [13] with the highest incidence among the middle age groups from our studies similar to some study [14] but differ from other [13]. Male to female ratio was found to be 2.5:1 with high predominance among males (72%). This is expected, as trauma is commoner in this group of patients similar to other series [5,13,14]. Both ears were almost equally affected in the ratio of 1.0:1.3 right to left, this could be associated with the fact that most assailants were right handed and likely that most of the acts of trauma such as slap occurred with the assailant and victims facing each other making the left ear to be predominantly affected compared to the right side. Some of the causes of overpressure include slap injuries and blast injuries. Slap injuries are extremely common and can be as a result of either a hand or water slap and these injuries usually result in a triangular or linear tear of the TM from previous study [12]. These slap injuries could be a product of fight, armed robbery attack as seen in our study however it was found to be commoner among the youths in more than 50%of cases reviewed and those in the adult were due to attack by the armed robbers or security agents. This was the highest cause of traumatic tympanic membrane perforation in our study compared to a similar study in other region of Nigeria where fight with spouse was the commonest aetiology recorded [13] which was the least in our study.

Slap from fights was the commonest cause of the traumatic perforation which was the commonest type of violence seen between individuals, mostly between security agents and the offender, then among students however other study found it resulting from marital conflict between wife and spouses [13] however the need to educate the students and security agents on other punitive measure as there is predisposition to conductive hearing loss or an imminent chronic suppurative otitis media if not properly managed. Slap was commoner among males than the females similar to other study [13], then road traffic injury most of which resulted from temporal bone fracture with cerebrospinal fluid (CSF) leakage with low frequency (15.6%) in our environment from the data presented, bleeding from the ear and damage to the ossicular attachment at the posterior-inferior part of the tympanic membrane with resultant conductive hearing loss of about 7-20dB [12] however our own study did not evaluate the degree of hearing loss from these traumatic perforation. The management protocol for skull base fracture with TM perforation/CSF leakage is masterly inactivity to avoid contamination with an ascending infection. Attempt at removing foreign body, self ear cleaning with variety of object cotton bud inclusive and wax removal in an unskilled manner either by the parents or the primary care physician with TM perforation was an important cause found mostly among the children similar to other reports [5,6,10,11], thus the need for a primary care physician to identify their limits with appropriate referral. Explosion is not a common phenomenon in our environment as it is relatively a peaceful one. Fall from a height with bleeding from the ear was also observed in ten months old child which further assessment showed perforated tympanic membrane.

Traumatic perforations often occur in the healthy members of the community; and generally the prognosis is excellent [6,8]. The two main factors that predispose to failure of the perforation to heal are loss of tissue and secondary infection. Secondary suppurative otitis media occurred in 37.5% of the ears in this series. This resolved with both antibiotic impregnated topical wick ear dressing and systemic antibiotics with healing of the perforations.

The most effective management is masterly inactivity. Because of the risk of introducing infection, the ear should not be cleaned out. The ear must be kept dry by preventing water from entering the ear canal [6,8]. If the perforation fails to close spontaneously by 3-6 months (in the absence of secondary infection), surgical closure is indicated [6,8]. If the perforation fails to close spontaneously in 3-6 months (in the absence of secondary infection), surgical closure is indicated [6,8]. However all our patient in this study had conservative, non touch technique or non-surgical treatment. The follow up was observed to be an average of thrice among the all the patients in the study. Healing with formation of neomembrane was observed only in five patients (7.8%) and it is among the under five's this is not surprising as they are still growing.

In conclusion traumatic perforation of the tympanic membrane is still common in our environment; affect all age groups, male more than the males, slap and RTI are the commonest aetiologies seen, left ear affected more than the right and sudden hearing loss is the commonest symptom of presentation. Even though it is not a common injury that is under-reported, there is the need to educate the student and security agents on alternative punitive measure, discourage the act of unskilled removal of foreign body, early identification, evaluation and referral of patients by primary care physician who saw these patients to reduce the attendant morbidity.

## Competing interests

The authors declare that they have no competing interests.

## Authors' contributions

AOA perform the conception and design of the study, or acquisition of data, or analysis and interpretation of data, ASK assisted in drafting the article and proof reading the manuscript. SS assisted in drafting the article or revising it critically for important intellectual content, ABS performed the final approval of the version to be submitted. All authors have read and approved the final manuscript
